# Transcriptional Profiling of the Rabbit Liver Infected With *Eimeria stiedae* Reveals Dynamic Host Cell Responses During the Induction and Resolution of Cholangitis

**DOI:** 10.1155/2024/4168719

**Published:** 2024-09-16

**Authors:** Miner Deng, Tianyi Hou, Yanting Wei, Wanting Zeng, Yaqiong Guo, Na Li, Lihua Xiao, Yaoyu Feng

**Affiliations:** ^1^ State Key Laboratory for Animal Disease Control and Prevention South China Agricultural University, Guangzhou 510642, China; ^2^ Guangdong Laboratory for Lingnan Modern Agriculture Center for Emerging and Zoonotic Diseases College of Veterinary Medicine South China Agricultural University, Guangzhou 510642, China

**Keywords:** cholangitis, *Eimeria stiedae*, host–pathogen interactions, liver, transcriptomics

## Abstract

*Eimeria stiedae* is one of the few eukaryotic pathogens that exclusively infect the liver and serves as a good model to study the host–pathogen interactions in this vital organ. In this study, we show that rabbits infected with *E. stiedae* develop severe but self-healing cholangitis. RNA-seq analysis of the liver gene expression landscapes over the long course of *E. stiedae* infection identified 912 differentially expressed genes (DEGs) in the prepatent period (794 up- and 118 downregulated genes), 2889 DEGs in the early oocyst shedding period (1870 up- and 1019 downregulated genes), 2859 DEGs in the peak oocyst shedding period (1923 up- and 936 downregulated genes), and 327 DEGs in the recovery period (164 up- and 163 downregulated genes). Combined with pathological observations, we identified dynamic changes in host–parasite interactions involving multiple pathways. They showed that *E. stiedae* infection induced full-blown inflammatory, Th1 and Th17 immune responses at all time points. This was associated with the strong innate immune responses during the prepatent period, including increased Toll-like and NOD-like receptor signaling. Despite mounting several damage control and repair responses, such as PI3K-Akt signaling, Ras signaling, and extracellular matrix-receptor interactions, the liver underwent severe metabolic dysfunction, oxidative damage, and coagulopathy after patency and at peak infection, possibly as a result of suppressed peroxisome activities and downregulated PPAR signaling. These responses largely disappeared during late infection, suggesting that the liver self-heals after severe cholangitis. These data provide new insights into host–pathogen interactions during *Eimeria* infection and improve our understanding of the pathogenesis of parasitic cholangitis.

## 1. Introduction

Cholangitisis an important clinicalmanifestation of infections by many parasites, such as *Cryptosporidium* spp. in immunocompromised persons and liver trematodes (*Opisthorchis* spp., *Clonorchis sinensis*, and *Fasciola hepatica*) and cestodes (*Echinococcus granulosus* and *E. multilocularis*) in humans and animals in many parts of the world [1]. Despite its public and veterinary health significance, the pathogenesis of parasitic cholangitis is poorly understood. Our understanding of the host–pathogen interactions in this area mostly come from in vitro studies conducted with *Cryptosporidium parvum* [2], which is an intestinal pathogen but also invades the biliary epithelium in immunocompromised individuals such as AIDS patients [3].

Among the 11 common *Eimeria* species in rabbits (*Oryctolagus cuniculus*), *E. stiedae* is the only one that invades biliary epithelial cells [4, 5] and is the most pathogenic species, causing cholangitis [6]. It, therefore, serves as a good model for studying parasitic cholangiopathy. Although some pathological examinations of rabbit livers infected with *E. stiedae* have been performed, we still have a poor understanding of the pathogenesis of cholangiohepatitis induced by the parasite [4, 6–9]. A previous study examined the transcriptomic landscape of several lifecycle stages of *E. stiedae* [10]. However, host cell responses to *E. stiedae* infection have thus far not been studied. As a hepatic *Eimeria* species, *E. stiedae* could induce unique host responses not seen in intestinal *Eimeria* infections. Transcriptomic studies of *E. stiedae* infection could also shed light on adaptive responses of the liver to other hepatic pathogens.

In this study, the transcriptomic responses in the liver of rabbits infected with *E. stiedae* were examined by RNA-seq analysis of tissue samples collected at the prepatent, early, peak, and late stages of oocyst shedding. The data obtained have revealed significant differences in host cell responses during the course of *E. stiedae* infection, leading to a better understanding of host immune responses and host–pathogen interactions during the induction and resolution of parasitic cholangitis.

## 2. Materials and Methods

### 2.1. Ethics Statement

The study was conducted in accordance with the Guide for the Care and Use of Laboratory Animals. The research protocol was approved by the Committee on the Ethical Use of Animals in Research, South China Agricultural University (No. 2021C051).

### 2.2. Animals and Parasites

Thirty 45-day-old New Zealand rabbits raised under coccidia-free conditions for the development of coccidial vaccines were used in this study. They were confirmed to be free of coccidia by microscopic analysis of fecal samples for *Eimeria* oocysts for three consecutive days before infection and 2, 5, and 8 days postinfection (DPI). The rabbits were randomly assigned to the infection and control groups. Each rabbit in the infection group was inoculated orally with 4 × 10^4^ sporulated oocysts of *E. stiedae*. The oocysts of *E. stiedae*, a gift from Foshan Standard Biotech Co. Ltd (Foshan, China), were previously passaged in four coccidia-free rabbits and allowed to sporulate in 2.5% potassium dichromate solution for 4 days. All rabbits used in this study were housed individually with free access to sterile water and food. Bodyweight was measured every 3 days.

### 2.3. Necropsy of Animals and Pathological Examinations of Liver

**Figure 1:**
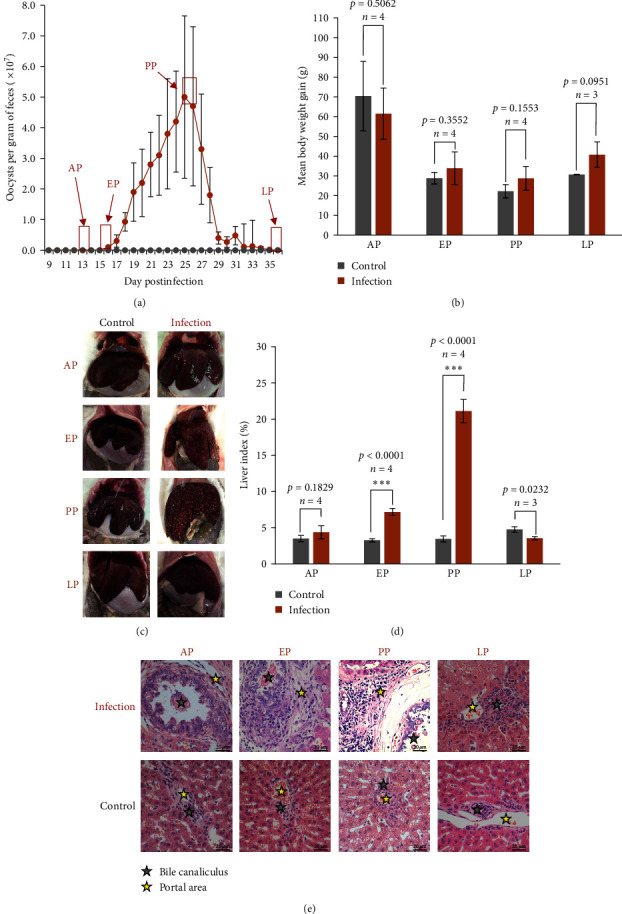
Morphological and pathological changes in the liver of rabbits infected with *Eimeria stiedae*. (a) Pattern of oocyst shedding in feces of rabbits infected with *E. stiedae*. Rabbits in the infection group were infected with 4 × 10^4^ oocysts, and oocyst shedding was measured every day after DPI 9. Three or four rabbits each in the infection and control groups were sacrificed at the prepatent (AP, at DPI 12), early (EP, at DPI 16, the beginning of oocyst shedding), peak (PP, at DPI 26), and late (LP, at DPI 36) oocyst shedding, with the liver being harvest for pathological and transcriptomic analysis. (b) The mean body weight gain of infection and control groups during the study. (c) Gross morphology of the liver of rabbits at different times of the *E. stiedae* infection. (d) Changes in liver index ( = liver weight/body weight × 100%) of rabbits during the *E. stiedae* infection course. (e) Pathological changes in the liver during *E. stiedae* infection. Scale bars, 20 μm.

At four time points during the course of infection, three to four rabbits in each group were sacrificed to collect the liver tissue and measure the liver index ( = liver weight/body weight × 100%), including the prepatent period at DPI 12 (the asexual period, also called AP, four rabbits each for infection and control per group), the beginning of oocyst shedding at DPI 16 (the early period, also called EP, four rabbits per group), peak oocyst shedding at DPI 26 (the peak period, also called PP, four rabbits per group), and the end of oocyst shedding at DPI 36 (the late period, also called LP, three rabbits per group) (Figure 1a). For histological analysis, the liver tissues were fixed in 4% paraformaldehyde for H&E and immunohistochemical (IHC) staining using conventional procedures. For IHC, antibodies against CD4^+^ (Servicebio, cat#GB11064, 1:500) and PLAU (Servicebio, cat#GB113137, 1:500) were used as the primary antibodies, a goat antirabbit antibody conjugated to horseradish peroxidase was used as the secondary antibody, and the 3,3′-diaminobenzidine (DAB) substrate (Abcam, cat#ab64261) was used to stain the host cell nuclei. The stained sections were examined using an Olympus BX53 microscope. Images were acquired using the CellSens Standard 1 software (Olympus) and processed using ImageJ and Photoshop. A fragment from each liver sample was immediately frozen in liquid nitrogen and stored at −80°C for RNA extraction and RNA-seq analysis.

### 2.4. RNA Isolation and Transcriptomic Sequencing

RNA was extracted from the 30 liver samples using TRIzol (Invitrogen, cat#15596026). RNA degradation was monitored using agarose gel electrophoresis, and the quality and quantity of RNA were assessed using a Nanodrop 8000 microspectrophotometer and an Agilent RNA ScreenTape Assay. The RNA preparations were delivered to the Annoroad Gene Technology Co., Ltd (Beijing, China) for library construction and sequencing on an Illumina NovaSeq 6000. The RNA-seq data generated in this study have been deposited in the SRA database of the National Center for Biotechnology Information under the accession number PRJNA1058682 (https://www.ncbi.nlm.nih.gov/sra/PRJNA1058682).

### 2.5. Mapping RNA-Seq Reads to Reference Genomes

Raw sequence reads were assessed for quality using FastQC v0.11.2. Adapter and low-quality sequences were trimmed using Trim galore v0.4.5a. The reference genome and annotations of *O. cuniculus* were downloaded from the genome website (https://ftp.ensembl.org/pub/release-108/fasta/oryctolagus_cuniculus/dna/), while an in-house genome assembly was used as the reference genome of *E. stiedae*. Clean sequence reads were mapped onto the reference genomes with default parameters by using STAR v2.7.6a. The alignment results were sorted and indexed using samtools v1.7.

### 2.6. Bioinformatics Analysis of Transcriptome Data

Expression matrices were generated using RSEM v1.3.3 analysis of the cleaned RNA-seq data. The “DESeq” package in R v4.1.0 was used to compare data from the infection and control groups at different oocyst-shedding periods. Differentially expressed genes (DEGs) were identified by absolute fold change >2 and *p*-values < 0.05. Principal component analysis (PCA) was used to assess the group differences, and the dot plots were generated by selecting the top two principal components. DEGs at different time points were illustrated with volcano plots using the “ggplot2” package.

Kyoto Encyclopedia of Genes and Genomes (KEGG) and Gene Ontology (GO) analyses were performed on DEGs between the infection and control groups at different oocyst-shedding periods using the DAVID database (https://david.ncifcrf.gov/). KEGG and GO term enrichment analyses were performed to identify biological pathways and terms involved, with *p*-values < 0.05 as the threshold for significant enrichment. Gene set enrichment analysis (GSEA) was used to determine the key KEGG pathways at peak oocyst shedding [11]. The enrichment score in GSEA was calculated by ranking the gene expression from the most to least significant.

The short time-series expression miner (STEM) was used to identify major temporal profiles of gene expression [12]. Log_2_ values (fold change) of DEGs were used as input files to obtain the profiles of gene expression over time.

### 2.7. Validation of RNA-Seq Results by Quantitative Reverse Transcription PCR (qRT-PCR)

Six DEGs were selected for confirmatory analysis of the RNA-seq results using qRT-PCR (Table S1). RNA preparations from different time points of the infection were used as the templates. Glyceraldehyde 3-phosphate dehydrogenase (GAPDH) was chosen as an endogenous reference gene. The primers used in the qRT-PCR are shown in Table S1. The qRT-PCR was performed on a Roche LightCycler 480 II using the HiScript II One Step qRT-PCR SYBR Green Kit (Vazyme, cat#Q221-01).

## 3. Results

### 3.1. Rabbits Infected with *E. stiedae* Experience Severe but Self-Resolving Cholangitis

After inoculation with *E. stiedae* oocysts, rabbits started to shed oocysts in feces at DPI 16 (Figure 1a). The oocyst output peaked approximately at DPI 26, and oocysts were not detected in the feces after DPI 36 (Figure 1a). Compared to those in the control group, rabbits in the infection group started to have rough hair, glazed eyes, and slow movement at DPI 10. The bodyweight gains of all rabbits showed no significant difference during the infection course (Figure 1b), but there were significant morphological differences in the liver between the two groups (Figure 1c,d). The liver of rabbits in the control group appeared normal (Figure 1c,d), while the liver of infected rabbits showed significant enlargement, numerous yellow lesions of various sizes, and a rough surface at PP (Figure 1c). In contrast, the liver collected from infected rabbits at EP showed significant enlargement but had only a small number of yellow lesions on the smooth surface (Figure 1c,d). However, those collected from infected rabbits at AP and LP remained largely normal (Figure 1c,d).

On histological analysis, the liver collected from infected rabbits at AP showed minor tissue damages, with enlarged biliary ducts, some inflammatory cells around the portal area, and the presence of some meronts in the epithelial cells (Figures 1e and S1). Inflammatory and immune responses were obviously increased in the portal area of the liver of infected rabbits at EP, when numerous gametocytes, zygotes, and unsporulated oocysts were found in the thickened and dilated biliary ducts (Figures 1e and S1). The livers from infected rabbits collected at PP were severely damaged, with lesions typical of sclerosing cholangitis and some necrosis of hepatic parenchymal cells (Figure 1e). The biliary ducts were filled with unsporulated oocysts and cell debris from the damaged columnar epithelia (Figures 1e and S1). There was also the formation of fibrous tissue, infiltration of numerous inflammatory and immune cells in the biliary ducts, periductal fibrosis, extracellular matrix (ECM) deposition, and granuloma formation (Figure 1e). These pathological changes in the liver largely disappeared at LP, with the biliary villi recovering and the number of inflammatory and immune cells decreasing (Figures 1e and S1). Although some areas of biliary tree stricture associated with fibrosis were observed, the overall histology of the liver from infected rabbits was similar to that of the control group (Figure 1e).

### 3.2. Rabbits Undergo Dynamic Transcriptomic Responses to Hepatic *E. stiedae* Infection

To explore host responses to *E. stiedae* infection in rabbits, RNA-seq analysis was performed on liver samples collected over the long infection course. Among the 18,807,009 to 20,110,347 cleaned sequence reads, 0%–10.47% mapped to the reference *E. stiedae* genome and 73.46%–90.09% mapped to the reference *O. cuniculus* genome (Table S2).

**Figure 2:**
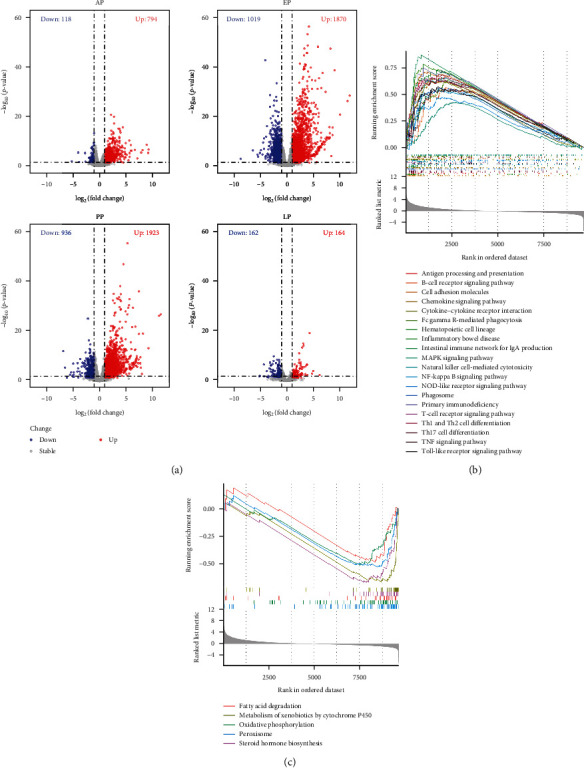
Dynamic changes in host gene expression after infection with *Eimeria stiedae*. (a) Volcano plots showing differential expression genes (DEGs) in the liver of rabbits infected with *E. stiedae* at the prepatent (AP), early (EP), peak (PP), and late (LP) oocyst-shedding periods. DEGs are shown as red (upregulated) or blue (downregulated) dots. Gene set enrichment analysis (GSEA) plots showing (b) upregulated and (c) downregulated KEGG pathways at PP. *p* < 0.05, log_2_ fold change >2.

Results of the PCA of the DEG data indicated that the infection and control groups had different transcriptomic responses at EP and PP, whereas the transcriptomic responses were more similar between the two groups at other time points. Compared with the control group, we identified 912, 2889, 2859, and 327 DEGs in the liver of rabbits collected at the prepatent, early, peak, and late oocyst shedding period, respectively (Figure 2a). Among them, 794, 1870, 1923, and 164 genes were upregulated, while 118, 1019, 936, and 163 genes were downregulated, respectively (Figure 2a).

To examine biological changes at different points, KEGG and GO enrichment analysis were used in significant DEGs (Figures S2 and S3). The KEGG pathway analysis showed that a variety of cytokines and immunity-related pathways were activated at AP and EP (Figure S2). These responses peaked at PP but mostly returned to normal at the end of oocyst shedding (Figure S2 and Table S3). The results of GO enrichment analysis supported this conclusion, with most upregulated genes being adaptive immunity and inflammation-related, with additional upregulated expression of innate immunity-related genes at AP (Figure S3). In contrast, most of the downregulated KEGG pathways and GO terms were mainly related to bile secretion, metabolism, and peroxisome and the associated PPAR signaling pathway, indicating the existence of extensive damages to liver functions by *E. stiedae* infection (Figures S2 and S3).

To further understand the process of host responses induced by *E. stiedae* infection, GSEA was performed to visually depict gene expression at PP (Figure 2b,c). The results obtained indicated that many inflammatory and immune-related signaling pathways were activated (Figure 2b), while the downregulated genes were mostly related to fatty acid degradation, metabolism of xenobiotics by cytochrome P450, oxidative phosphorylation, peroxisome and steroid hormone biosynthesis pathways (Figure 2c).

**Figure 3:**
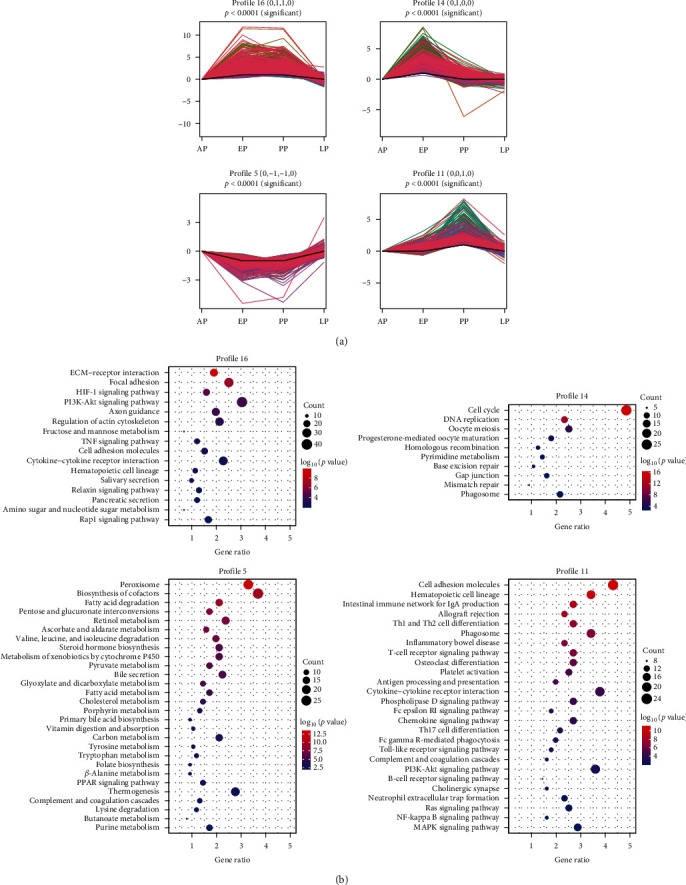
Transcriptomic patterns of differential expression genes (DEGs) at the prepatent (AP), early (EP), peak (PP), and late (LP) periods. (a) Short time-series expression miner (STEM) analysis showing four major DEG profiles across four periods (*p* < 0.01). (b) Scatter diagrams showing the KEGG pathways enriched in these four profiles. The *x*-axis represents the ratio of DEG, while the *y*-axis indicates the enriched KEGG pathways.

In addition, to identify the dynamic changes in gene expression during *E. stiedae* infection, STEM was used to examine the dynamic changes in transcriptomic responses over the infection course (Figure 3). Among the 19 profiles identified, four were significant, including profiles 16, 5, 14, and 11 (Figure 3a). Profile 5 indicates metabolism and oxidative responses were suppressed at EP and PP, while Profile 16 indicates that ECM-receptor interaction was activated during the same periods (Figure 3b). In contrast, Profile 14 indicates that cell cycles and DNA replications were activated during EP, possibly in response to tissue damages caused by the emerging parasites (Figure 3b). In addition, genes in profile 11 were mainly activated during PP and were associated with immune and inflammatory responses (Figure 3b).

Six DEGs were randomly selected for verification of the transcriptomic responses by qRT-PCR. The correlation coefficient between the RNA-seq and qRT-PCR data reached 0.97 (*R*^2^ = 0.94, *p* < 0.001), indicating that the results of the RNA-seq analysis in this study were reliable.

### 3.3. Rabbits Mount Early Innate Immune Responses During *E. stiedae* Infection

**Figure 4:**
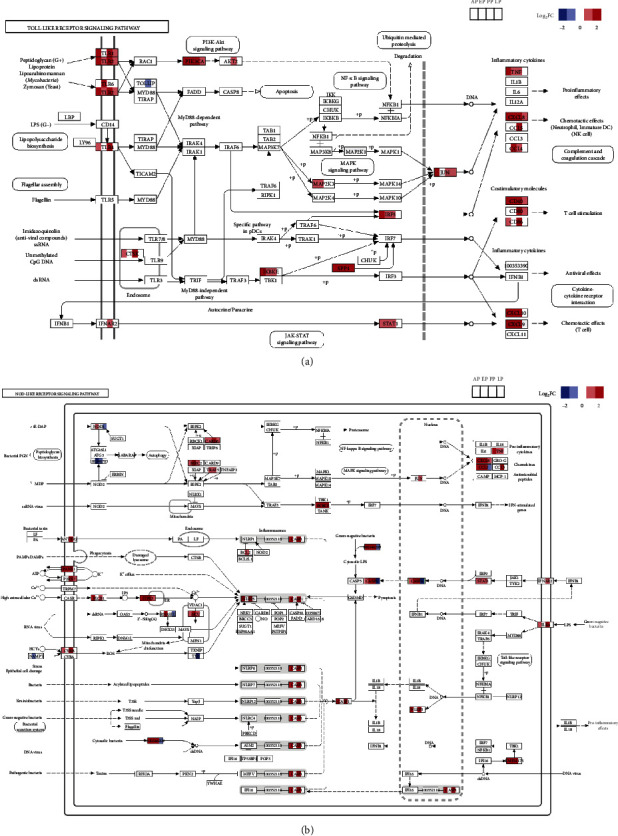
Dynamic transcriptomic responses of pattern recognition receptors (PRRs) during *Eimeria stiedae* infection. The pathway diagrams show differentially expressed genes (DEGs) in (a) Toll-like and (b) NOD-like receptor signaling pathways at the prepatent (AP), early (EP), peak (PP), and late (LP) oocyst-shedding periods. The upregulated (red) or downregulated (blue) expression of key PRR genes at different periods is indicated.

Both GO analysis and KEGG analysis revealed that innate immune response was highly enriched at AP (Figures S2 and S3). Several pathways related to innate immune responses (including Toll-like and NOD-like receptor pathways and natural killer-mediated cytotoxicity) and their downstream pathways (such as NF-*κ*B signaling pathway and MAPK pathway) were upregulated at AP (Figure S2). As many key elements (including TLR1, TLR2, TLR4, CD86, CASP1, and CTSK) showed high expression at AP, these innate immune responses probably represented a host defense strategy against the invading pathogen (Figures 4 and S4). Toll-like and NOD-like receptor signaling responses were maintained at EP and PP (Figures S4a and S4b), but no enrichment in innate immune responses was observed in both GO and KEGG analyses at subsequent time points (Figures S2 and S3).

To further understand the responses of pattern recognition receptors (PRRs) during *E. stiedae* infection, DEGs involved in Toll-like and NOD-like receptor signal pathways were analyzed in more detail (Figure 4). In the Toll-like receptor signaling pathway, members of the TLR2 family (such as TLR1, TLR2, and TLR6) as well as TLR4 were significantly upregulated (Figure S4a). As a result, the downstream PI3K-Akt, MAPK, and NF-*κ*B signaling pathways were upregulated, leading to the activation of the JAK-STAT signaling pathway and the generation of inflammatory cytokines and chemokines and T-cell stimulation (Figures 4a and S4c). As an upstream pathway of the NF-*κ*B and MAPK pathways, some key members of the MyD88-independent signaling pathway (including TRAF3 and SPP1) were upregulated (Figures 4a and S4a). In addition, the NOD-like receptor signal pathway also contributed to the activation of the NF-*κ*B and MAPK signaling pathways, producing proinflammatory cytokines and chemokines during *E. stiedae* infection (Figures 4b and S4b). These results suggested that both the Toll-like and NOD-like receptor signaling pathways were activated to defend against *E. stiedae* infection.

Upon activation of PRRs, inflammasomes became activated, which in turn trigger many antipathogen inflammatory responses (Figure 4). Key components of the NLRP3 inflammasome (including NLRP3, CARD6, and CASP1) were upregulated during *E. stiedae* infection, mediating caspase-1-dependent processing and activation of IL-1*β* and IL-18 (Figures 4b and S4b). PANX1 and P2X7, two essential components of acute inflammatory responses for cytokine production, were upregulated during the development of *E. stiedae* (Figure 4b). Interestingly, although many key components involved in pyroptosis (including NLRP3, CASP1, CASP5, PANX1, and P2X7) were upregulated, the expression of the key element GSDMD remained unchanged during the course of *E. stiedae* infection (Figure 4b). This suggests that *E. stiedae* was unlikely to induce pyroptosis of hepatic cells.

### 3.4. Th1 and Th17 Responses Are Activated in the Liver During Active *E. stiedae* Infection

**Figure 5:**
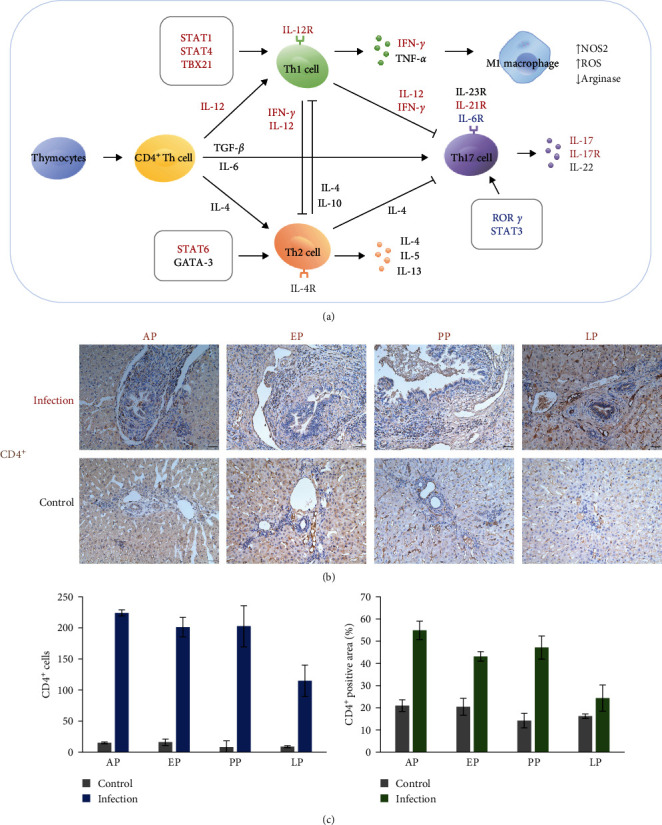
Activation of CD4^+^ Th cell in the liver of rabbits infected with *Eimeria stiedae*. (a) Summary of CD4^+^ Th cell differentiation at the prepatent (AP), early (EP), peak (PP), and late (LP) oocyst-shedding periods. Genes marked red have upregulated expression, while those marked blue have downregulated expression. (b) Immunohistochemistry of CD4 expression in the liver collected at different time points. Scale bars, 50 μm. Changes in the (c) number and (d) percentage of CD4^+^ cells during *E. stiedae* infection based on Image J analysis of the immunohistochemistry data.

To understand the adaptive immune responses to *E. stiedae*, the dynamics of major signaling pathways involved in adaptive immune responses were examined. GO term analysis showed that adaptive immune response was enriched at both AP and PP, while KEGG analysis indicated that Th1, Th2, and Th17 cell differentiation were upregulated during *E. stiedae* infection, suggesting that adaptive immune responses were induced at all time points (Figures S2 and S3). STEM analysis showed that many immune pathways (such as Th1 and Th2 cell differentiation, T-cell receptor signaling pathway, antigen processing and presentation, cytokine-cytokine receptor interaction, intestinal immune network for IgA production, and Th17 cell differentiation) were enriched in Profile 11, indicating the occurrence of strong adaptive immune responses at PP (Figure 3b and Table S4). IHC analysis confirmed that the number of CD4^+^ T cells was increased at different time points (Figure 5b,c).

To further characterize the adaptive immune responses of rabbits infected with *E. stiedae*, the expression of genes involved in CD4^+^ T cell differentiation was examined in more detail (Figures 5a, S4c, and S5). Key genes involved in Th1 cell differentiation (including STAT1, STAT4, TBX21, IL-12R, IL-12, and IFN-*γ*) were upregulated, while most genes involved in Th2 cell differentiation (such as GATA-3, IL-4, IL-4R, IL-5, IL-10, and IL-13) showed no significant changes during *E. stiedae* infection, suggesting that *E. stiedae* induced mainly Th1 type immune responses (Figures 5a, S4c, and S5a). Many genes related to Th17 cell differentiation (including IL-17, IL-17R, and IL-21R) were upregulated, suggesting that *E. stiedae* had further induced Th17-type immune responses (Figures 5a, S4c, and S5b). In contrast, the essential genes involved in Treg responses (including FOXP3, TGF-*β*, and IL-10) remained unchanged during the course of *E. stiedae* infection, suggesting that *E. stiedae* is unlikely to induce changes in the numbers of Treg cells in the liver (Figures 5a, S4c, and S5b).

### 3.5. Oxidative Damages, Metabolic Disorders, and Coagulopathy Occur in the Liver of Rabbits During Active *E. stiedae* Infection

**Figure 6:**
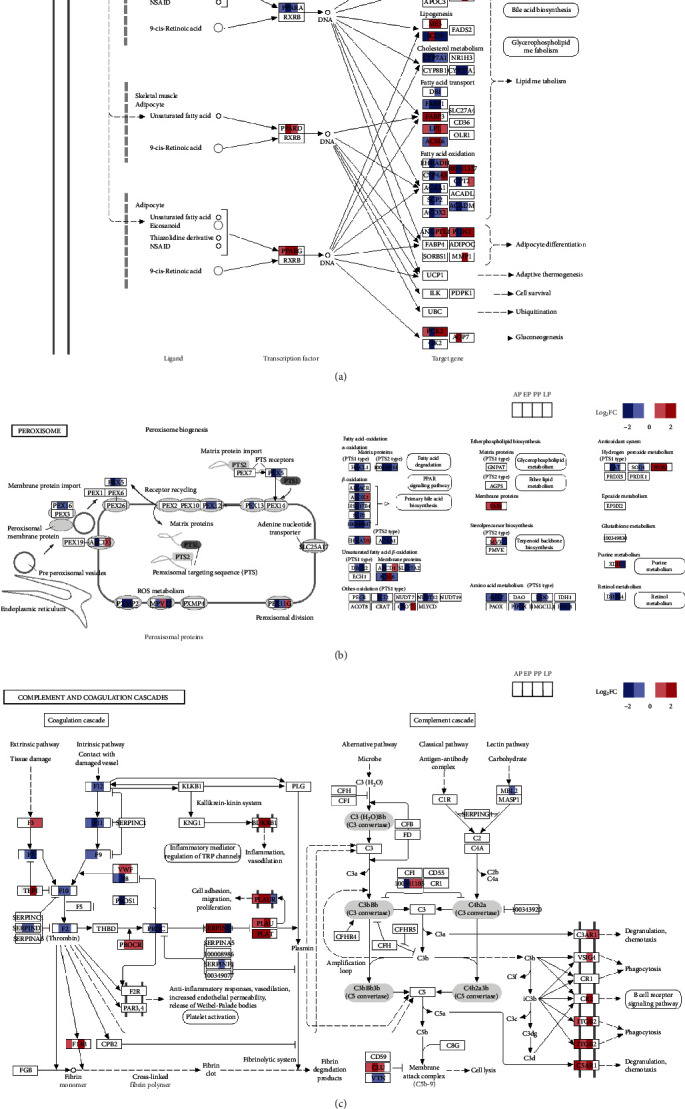
Transcriptomic changes in the peroxisome proliferator-activated receptor (PPAR) signaling pathway, the peroxisome pathway, and the complement and coagulation cascades at the prepatent (AP), early (EP), peak (PP), and late (LP) oocyst-shedding periods. (a) The PPAR signaling pathway is the peroxisome pathway. (b) The peroxisome pathway. (c) The complement and coagulation cascades. The upregulated (red) or downregulated (blue) expression of key genes at different periods is indicated.

The KEGG, GO, and STEM analyses of the RNA-seq data all showed that lipid metabolisms (such as fatty acid degradation, fatty acid metabolism, primary bile acid biosynthesis, and steroid hormone biosynthesis), carbohydrate metabolisms (such as carbon metabolism, pentose, and glucuronate interconversions, ascorbate and aldarate metabolism, pyruvate metabolism, glyoxylate and dicarboxylate metabolism, and butanoate metabolism), amino acid metabolism (including valine, leucine and isoleucine degradation, tyrosine metabolism, tryptophan metabolism, and lysine degradation), metabolisms of cofactors and vitamins (including biosynthesis of cofactors, vitamin digestion and absorption, retinol metabolism, porphyrin metabolism, and folate biosynthesis), functions of the liver (such as cholesterol metabolism, complement and coagulation cascades, and bile secretion), and oxidative responses (including peroxisome) were significantly suppressed at EP and PP (Figure 3b and Table S4). The reduction in metabolism was likely the result of the concurrent downregulation of the PPAR (peroxisome proliferator-activated receptor) signaling pathway, which regulates liver metabolism (Figure 6a). In particular, the expression of the major transcription factor in the liver, PPAR*α*, was significantly downregulated during *E. stiedae* infection (Figure 6a). In contrast, PPAR*δ* and PPAR*γ*, two other key transcription factors regulating lipid metabolism in the skeletal muscle and adipocytes, were adaptively upregulated at EP and PP (Figure 6a). As a result, lipid metabolism (including fatty acid degradation and bile acid biosynthesis) was suppressed in the liver during *E. stiedae* infection (Figures 6a, S2, and S3).

Nearly all of the genes involved in the peroxisome pathway were downregulated at EP and PP, indicating the inhibition of peroxisomal catabolic and anabolic metabolisms (Figure 6b). Several essential factors for the peroxisomal biogenesis, such as PEX1, PEX3, PEX5, PEX7, PEX12, and PEX16, were downregulated at EP and PP (Figure 6b). The key elements of fatty acid-oxidation in the peroxisome (such as ACOX2, HSD17B4, and SCP2) were also downregulated during *E. stiedae* infection, indicating that primary bile acid biosynthesis was suppressed and liver functions were impaired (Figure 6b).

The liver damage during *E. stiedae* infection was associated with impaired complement and coagulation responses (Figure 6c). Many coagulation factors (including F2, F5, F7, F8, F9, F10, F11, and F12) were downregulated, while serine proteases PLAT (plasminogen activator, tissue type) and PLAU (plasminogen activator, urokinase) were upregulated, suggesting that *E. stiedae* infection impaired coagulation and induced excessive fibrinolysis (Figures 6c and S6). In addition, although MBL2, MASP1, CFB, and CFHR5 were downregulated, the expression of most genes involved in the complement cascade showed no significant changes during *E. stiedae* infection. Some downstream genes of the complement cascade pathway (including C8G, C3AR1, VSIG4, ITGB2, and C5AR1) were upregulated, indicating that the complement system was activated to defend against *E. stiedae* infecting the liver. Moreover, the platelet activation pathway was also significantly activated at PP, supporting the occurrence of coagulation disorder in the liver during *E. stiedae* infection (Figures 1e, 3b, and Table S4).

The expression of most genes involved in the metabolism function and oxidative stress returned to normal at the end of *E. stiedae* infection (Figure 6a,b). Some of the genes (such as APOA1, ME3, FABP3, and FABP4) were upregulated at PP and LP, suggesting the occurrence of compensatory responses to hepatic damages (Figure 6a,b).

### 3.6. The Liver Self-Heals During *E. stiedae* Infection Is Associated with Upregulation in PI3K/Akt, Ras, and ECM–Receptor Interaction Pathways

The PI3K/Akt signaling pathway was activated during *E. stiedae* infection, possibly as a result of the upregulation of Toll-like, B-cell receptor JAK/STAT, focal adhesion, and chemokine signaling pathways as described (Figure S7a). Many key genes of the PI3K/Akt signaling pathway were upregulated, such as PIK3CA, PIK3R5, AKT2, and BCL2 (Figure S7a). This result indicated that the activated PI3K/Akt signaling pathway promoted anti-apoptotic effects and cell growth to protect against the damage caused by *E. stiedae* infection (Figure S7a).

Many DEGs associated with the RAS signaling pathway (such as GAB1, SHC1, ZAP70, LAT, GNB5, TIAM1, RAC1, and KSR2) were also upregulated in the liver infected with *E. stiedae* (Figure S7b). The upstream pathways (including the T-cell receptor signaling pathway and long-term potentiation) of the RAS pathway were activated, inducing the expression of genes involved in the actin cytoskeleton, MAPK, and calcium signaling pathways (Figure S7b). Therefore, RAS responses in the liver might regulate inflammation, fibrosis, cell growth, and survival to promote liver recovery from *E. stiedae* infection.

ECM–receptor interaction, which contributes to sclerosing cholangitis and hepatic fibrosis, was activated at EP and PP as described above (Figure 3b). The expression trend of these genes during *E. stiedae* infection is depicted in Figure S11, showing that many genes associated with ECM–receptor interaction (such as COL1A2, LAMB2, SPP1, TNC, NPNT, and FRAS1) were significantly regulated in the liver infected with *E. stiedae* (Figure S8). This was in agreement with the appearance of the excessive ECM seen in H&E analyses of the liver (Figure 1e).

## 4. Discussion

In this study, the dynamic changes in host responses are described during the induction and resolution of cholangitis in the liver of rabbits infected with *E. stiedae*. Transcriptomic analysis reveals that *E. stiedae* infection significantly activates innate immune responses during AP, which turns on inflammatory, Th1 and Th17 immune responses at all time points. Despite mounting several damage control and repair responses such as PI3K-Akt signaling, Ras signaling, and ECM-receptor interactions, the liver undergoes severe metabolic disorders, oxidative damages as the result of suppressed peroxisome activities, and coagulopathy after patency and at PP. These responses have largely disappeared in late infection, suggesting that the liver is largely self-healing after *E. stiedae* infection except for the apparent presence of biliary fibrosis.

The liver infected with *E. stiedae* shows high regenerative potential, undergoing compensatory hyperplasia to recover and ensure hepatic functions. Although a previous study has demonstrated reduced body mass and fat levels in rabbits infected with *E. stiedae* [13], the body weight gains of all infected rabbits have shown no significant difference in this study (Figure 1b), which may be due to the liver enlargement during *E. stiedae* infection (Figure 1c,d). The liver is known to expand and recover to maintain its function and metabolic needs after injury or excision [14, 15]. In our study, compensatory hyperplasia and self-recovery of the liver are apparent during *E. stiedae* infection. Unlike other parasites invading the liver or bile ducts that can cause chronic or repeated infections [16], *E. stiedae* completes the entire life cycle in approximately 1 month, and the host eliminates all the parasites and recovers rapidly. Currently, we still have a poor knowledge of the liver-*E. stiedae* interactions, but data on host responses to the hepatic coccidiosis provide new insights into the liver repair mechanisms against parasitic infection and the induction and recovery of cholangitis.

Innate immune responses upon *E. stiedae* infection appear to model host inflammatory and immune responses to the parasite. KEGG and GO analyses show that innate immune responses, inflammatory responses, Toll-like and NOD-like receptor signaling are significantly upregulated during *E. stiedae* infection (Figures S2 and S3), which is consistent with the suggestion that the innate immune system functions as the first line of host defense against pathogens [17]. Toll-like and NOD-like receptor signaling pathways are also upregulated in the liver infected with *T. gondii* [18, 19], consistent with the suggestion that TLRs play a central role in the pathogen clearance [17]. Interestingly, a previous study suggested that acute *T. gondii* infection may promote liver injury via TLR2 and TLR4 signaling pathways [20].

*E. stiedae* infection apparently stimulates strong Th1- and Th17-type immune responses. Transcriptomic analysis suggests that the Th1 response is increased in the infected liver as a result of the upregulated IL-12 and IFN-*γ* expression. IL-12 has been shown to be associated with protection against *T. gondii* infection by stimulating the proliferation of NK cells and CD4 T cells to produce massive IFN-*γ* [19]. In addition, the expression of many genes related to Th17 cell differentiation is upregulated (Figures 5a, S4c, and S5b), which is consistent with recent findings on the involvement of Th17 responses in the control of other parasitic infections [21, 22]. These Th1 and Th17 responses start early in *E. stiedae* infection, climax at PP, and return to normal during the recovery from cholangitis. This suggests that host immune responses may play an important role in the pathogenesis of sclerosing cholangitis induced by *E. stiedae* infection.

Damages of the liver in *E. stiedae* infection are probably mediated by mechanic injuries during parasite maturation in the biliary tree and by the downregulation of the peroxisome pathway. Extensively tissue damages and necrosis of the biliary epithelia were observed during patent and peak infection with *E. stiedae*, due to the rapid proliferation of the parasites and the indirect effect of the excessive immunological responses to *E. stiedae* infection. As a result, the biliary tree is obstructed by oocysts and epithelial desquamation, leading to cholangitis, biliary obstruction, and cholestasis. The latter has been observed in previous studies [5]. Hepatic cells probably also undergo degeneration due to reduced metabolism and nutrient absorption in the infected liver [4]. In addition, peroxisomes regulate a variety of essential metabolic activities and sequester diverse oxidative reactions; therefore, play an important role in cellular metabolism and reactive oxygen species (ROS) detoxification [23, 24]. The reduced peroxisomal activity seen in *E. stiedae* infection may trigger oxidative stress in cells, resulting in increased ROS production and liver damages [23]. Previously, it has been shown that peroxisomes are hijacked by some intracellular pathogens to evade elimination by the host immune system [25–27]. Therefore, the downregulated peroxisome pathway in the liver may be a potential strategy of *E. stiedae* to promote its survival in an organ rich in oxygen and free radicals.

The metabolic dysfunctions are likely the result of the concurrent downregulation of the PPAR*α* signaling pathway due to the suppression of the peroxisome activities by *E. stiedae*. Previous studies demonstrated that the PPAR*α* pathway is a major response downstream of the peroxisome pathway, regulating essential metabolic processes in the liver [28]. Our transcriptomic analysis indicates that the PPAR*α* and peroxisome pathways are downregulated during infection, along with many metabolic pathways of hosts (such as fatty acid degradation, bile acid biosynthesis, and glycerophospholipid metabolism). The downregulation of metabolic pathways also occurs in the liver infected with other parasites [29–33]. Interestingly, PPAR*α* has been reported as a potential strategy to counteract fibrosis [34], contributing to the ECM deposition during EP and PP and liver fibrosis during late *E. stiedae* infection. In addition, the liver infected with *E. stiedae* has imbalanced complement and coagulation responses, which have been observed in the liver infected with other pathogens [31, 35, 36].

The self-recovery during *E. stiedae* infection is probably mediated by several host signaling pathways. Many genes involved in PI3K/Akt, Ras, and ECM-receptor interaction pathways are upregulated during the *E. stiedae* infection course, suggesting that these singling pathways facilitate the liver self-healing and restoration of hepatic functions. The liver becomes largely normal at the end of oocyst shedding, with some residual fibrosis. The mechanism of liver self-healing in *E. stiedae* infection should be studied further for the development of new therapies against hepatopathy.

## 5. Conclusions

In conclusion, the results of histopathologic observations and RNA-seq analysis have revealed dynamic host cell responses in the liver infected with *E. stiedae*. The host mounts significant innate, Th1, and Th17 immune responses early in the prepatent period, which persists throughout the remainder of the *E. stiedae* infection. The liver undergoes severe metabolic dysfunction, oxidative damage, and coagulopathy. These findings improve our understanding of host–pathogen interactions during *E. stiedae* infection and the pathogenesis of cholangitis. They facilitate the development of new therapies for cholangitis and liver fibrosis.

## Data Availability

The RNA-seq data reported in this paper are available in the SRA database of the National Center for Biotechnology Information under the accession number PRJNA1058682 (https://www.ncbi.nlm.nih.gov/sra/PRJNA1058682).

## Supporting Information

Supporting Information Additional supporting information can be found online in the Supporting Information section at the end of this article. Figure S1: histological changes of the liver infected with *Eimeria stiedae* at the prepatent (AP), early (EP), peak (PP), and late (LP) oocyst-shedding periods. Both pathological changes and parasites at different lifecycle stages are observed in H&E-stained liver sections. Green arrows indicate trophozoites and schizonts, yellow arrows indicate gametocytes and blue arrows indicate zygotes and unsporulated oocysts. Figure S2: global transcriptomic responses of the liver infected with *Eimeria stiedae* using KEGG pathway analysis. The scatter diagrams show the major upregulated (left) and downregulated (right) KEGG pathways at the prepatent (AP), early (EP), and peak (PP) oocyst-shedding periods. The *x*-axis represents the ratio of differentially expressed genes (DEGs), and the *y*-axis indicates enriched KEGG pathways. Figure S3: dynamic transcriptomic changes of enriched GO terms in the liver infected with *Eimeria stiedae* at different periods. The bar plots show major upregulated (left) and downregulated (right) GO terms at the prepatent (AP), early (EP), and peak (PP) oocyst-shedding periods. The *x*-axis represents the ratio of differentially expressed genes (DEGs), and the *y*-axis indicates the enriched GO term. Figure S4: expression changes of differentially expressed genes (DEGs) involved in innate and adaptive immune responses during *Eimeria stiedae* infection. Heatmaps of DEGs involved in (a) Toll-like receptor signaling pathway, (b) NOD-like receptor signaling pathway, and (c) CD4+ Th cell subset differentiation at the prepatent (AP), early (EP), peak (PP), and late (LP) oocyst-shedding periods, with red indicating upregulated expression and blue indicating downregulated expression. Figure S5: dynamic adaptive immune responses of the liver infected with *Eimeria stiedae*. Expression trend of differentially expressed genes (DEGs) involved in (a) Th1 and Th2, and (b) Th17 cell differentiation at the prepatent (AP), early (EP), peak (PP), and late (LP) oocyst-shedding periods. Genes marked red have upregulated expression, while those marked blue have downregulated expression. Figure S6: expression changes of PLAU at different periods of *Eimeria stiedae* infection, based on immunohistochemistry. (a) Immunohistochemistry analysis of PLAU expression in the liver of rabbits collected at the prepatent (AP), early (EP), peak (PP), and late (LP) oocyst-shedding periods. Scale bars, 50 μm. The (b) number and (c) percentage of positive cells were quantified using the Image J software. Figure S7: transcriptomic changes of differentially expressed genes (DEGs) involved in the PI3K-Akt and Ras signaling pathways in the liver during *Eimeria stiedae* infection. Expression trend of DEGs involved in (a) PI3K-Akt and (b) Ras signaling pathways in the liver infected with *E. stiedae* at the prepatent (AP), early (EP), peak (PP), and late (LP) oocyst-shedding periods. Genes marked red have upregulated expression, while those marked blue have downregulated expression. Figure S8: expression changes of differentially expressed genes (DEGs) involved in the ECM-receptor interaction in the liver at the prepatent (AP), early (EP), peak (PP), and late (LP) oocyst-shedding periods. Genes marked red have upregulated expression, while those marked blue have downregulated expression. Table S1: primers used in qRT-PCR to validate the RNA-seq results. Table S2: summary of sequencing reads mapping to the reference genome and quality parameters. Table S3: KEGG enrichment among the upregulated genes in different periods. Table S4: summary of STEM significantly clustered profiles.
