# Supplementary material for: Transcriptional Profiling of the Rabbit Liver Infected With Eimeria stiedae Reveals Dynamic Host Cell Responses During the Induction and Resolution of Cholangitis
Source: Transbound Emerg Dis. 2024 Sep 16;2024:4168719. doi: 10.1155/2024/4168719 (PMC12017222; doi:10.1155/2024/4168719)
Supplement: Supporting Information — Additional supporting information can be found online in the Supporting Information section at the end of this article. Figure S1: histological changes of the liver infected with Eimeria stiedae at the prepatent (AP), early (EP), peak (PP), and late (LP) oocyst-shedding periods. Both pathological changes and parasites at different lifecycle stages are observed in H&E-stained liver sections. Green arrows indicate trophozoites and schizonts, yellow arrows indicate gametocytes and blue arrows indicate zygotes and unsporulated oocysts. Figure S2: global transcriptomic responses of the liver infected with Eimeria stiedae using KEGG pathway analysis. The scatter diagrams show the major upregulated (left) and downregulated (right) KEGG pathways at the prepatent (AP), early (EP), and peak (PP) oocyst-shedding periods. The x-axis represents the ratio of differentially expressed genes (DEGs), and the y-axis indicates enriched KEGG pathways. Figure S3: dynamic transcriptomic changes of enriched GO terms in the liver infected with Eimeria stiedae at different periods. The bar plots show major upregulated (left) and downregulated (right) GO terms at the prepatent (AP), early (EP), and peak (PP) oocyst-shedding periods. The x-axis represents the ratio of differentially expressed genes (DEGs), and the y-axis indicates the enriched GO term. Figure S4: expression changes of differentially expressed genes (DEGs) involved in innate and adaptive immune responses during Eimeria stiedae infection. Heatmaps of DEGs involved in (a) Toll-like receptor signaling pathway, (b) NOD-like receptor signaling pathway, and (c) CD4+ Th cell subset differentiation at the prepatent (AP), early (EP), peak (PP), and late (LP) oocyst-shedding periods, with red indicating upregulated expression and blue indicating downregulated expression. Figure S5: dynamic adaptive immune responses of the liver infected with Eimeria stiedae. Expression trend of differentially expressed genes (DEGs) involved in (a) Th1 an [file 4168719.f1.pdf]

Supplementary Figure

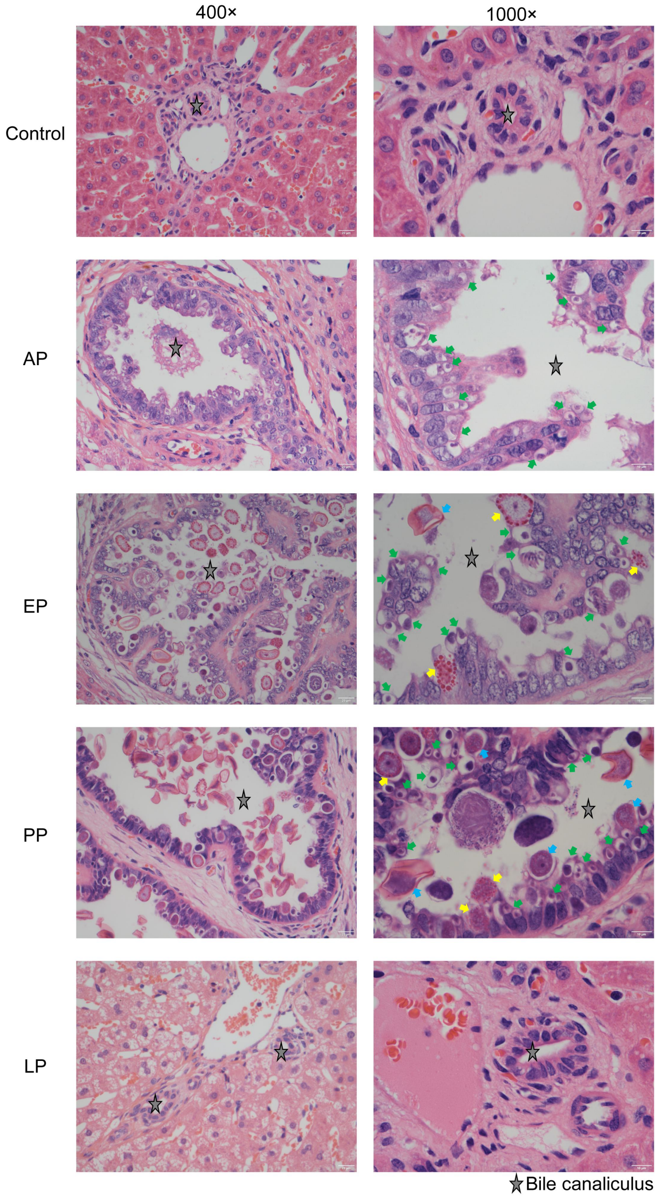

Figure S1: Histological changes of the liver infected with *Eimeria stiedae* at the prepatent (AP), early (EP), peak (PP), and late (LP) oocyst-shedding periods. Both pathological changes and parasites at different lifecycle stages are observed in H&E stained liver sections. Green arrows indicate trophozoites and schizonts, yellow arrows indicate gametocytes, and blue arrows indicate zygotes and unsporulated oocysts.

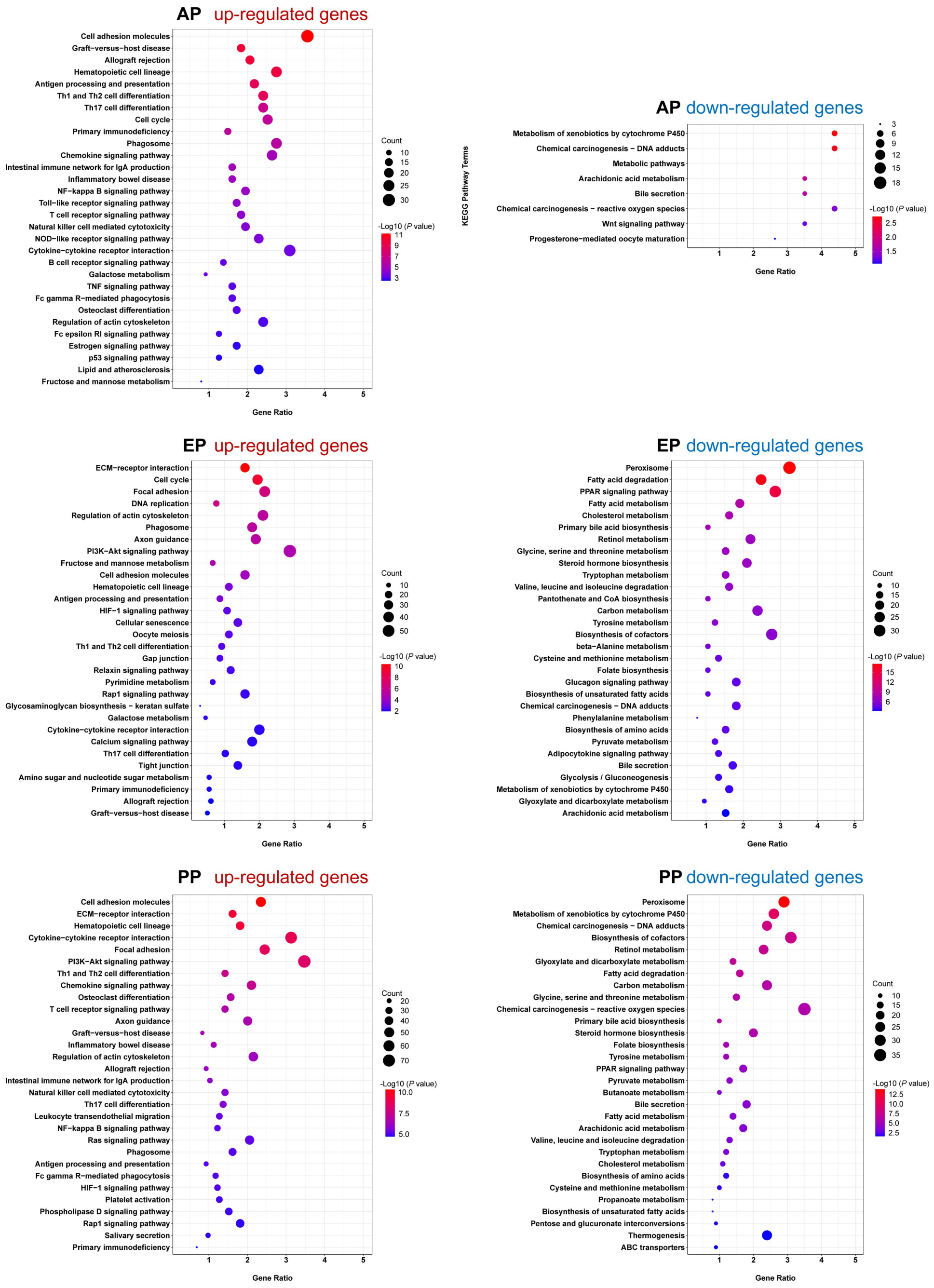

Figure S2: Global transcriptomic responses of the liver infected with *Eimeria stiedae* using KEGG pathway analysis. The scatter diagrams show the major up-regulated (left) and down-regulated (right) KEGG pathways at the prepatent (AP), early (EP) and peak (PP) oocyst-shedding periods. The x-axis represents the ratio of differentially expressed genes (DEGs) and the y-axis indicates enriched KEGG pathways.

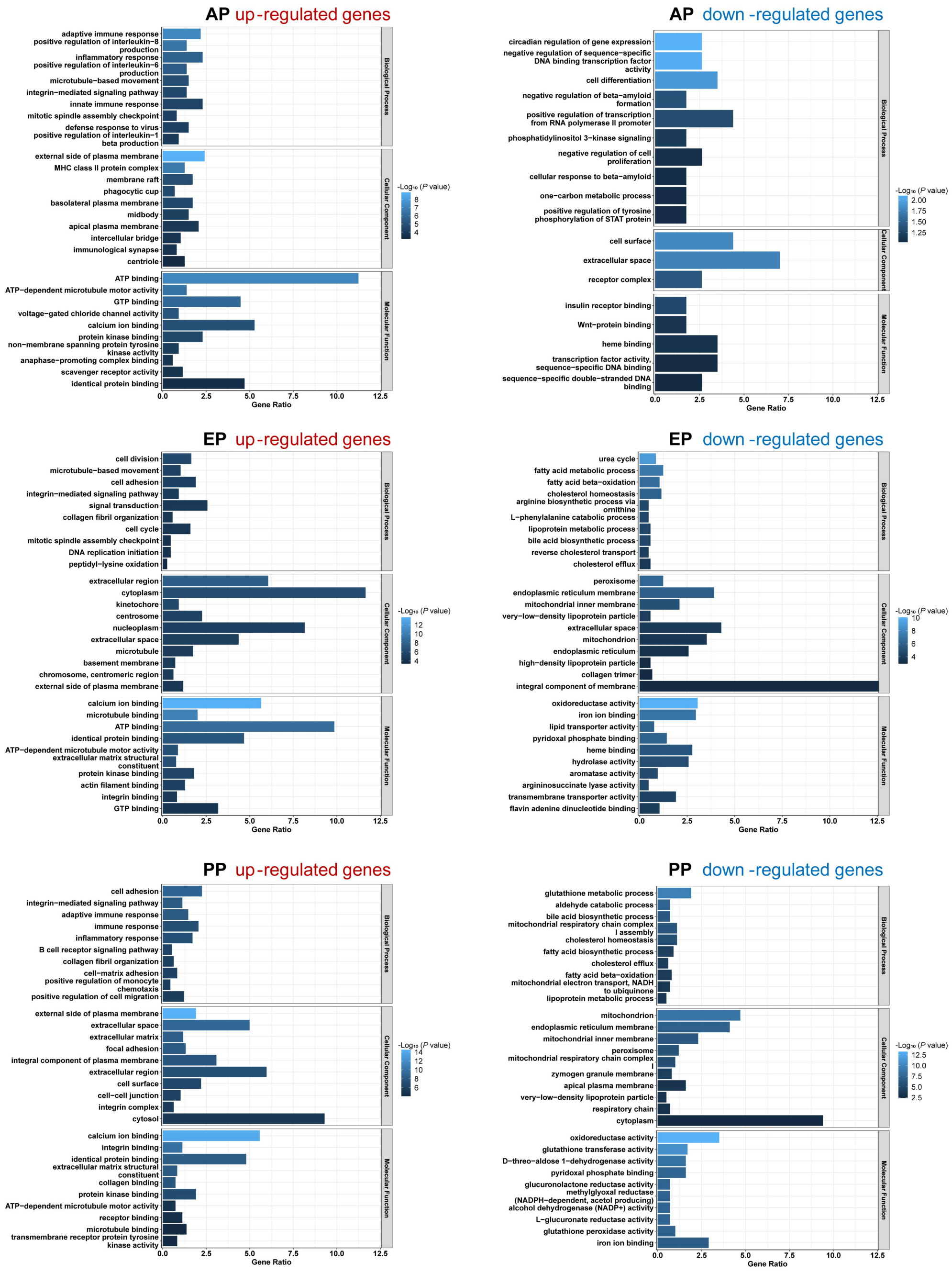

Figure S3: Dynamic transcriptomic changes of enriched GO terms in the liver infected with *Eimeria stiedae* at the prepatent (AP), early (EP) and peak (PP) oocyst-shedding periods. The bar plots show major up-regulated (left) and down-regulated (right) GO terms at the prepatent, early and peak oocyst-shedding. The x-axis represents the ratio of differentially expressed genes (DEGs) and the y-axis indicates the enriched GO term.

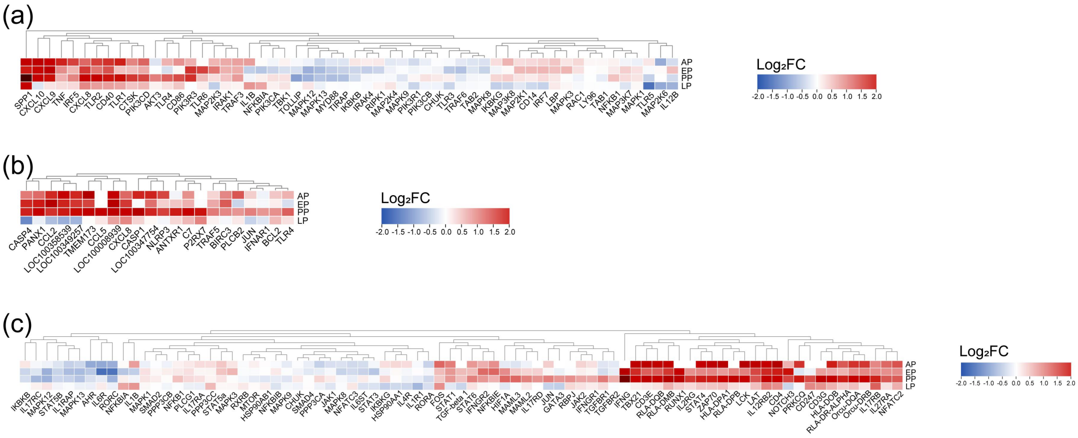

Figure S4: Expression changes of differentially expressed genes (DEGs) involved in innate and adaptive immune responses during *Eimeria stiedae* infection. Heatmaps of DEGs involved in (a) Toll-like receptor signaling pathway, (b) NOD-like receptor signaling pathway, and (c) $CD4^{+}$ Th cell subset differentiation at the prepatent (AP), early (EP), peak (PP), and late (LP) oocyst-shedding periods, with red indicating up-regulated expression and blue indicating down-regulated expression.

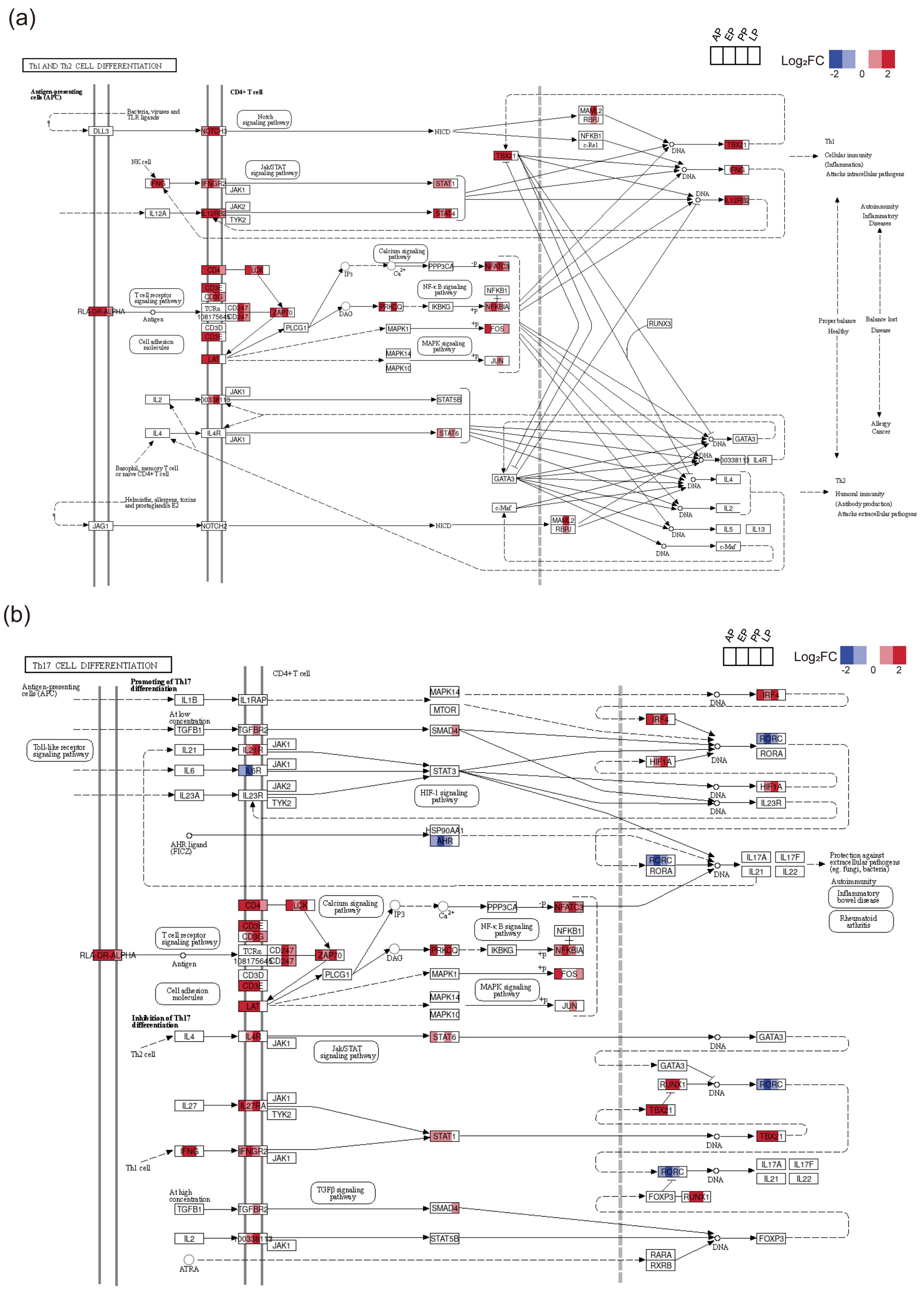

Figure S5: Dynamic adaptive immune responses of the liver infected with *Eimeria stiedae*. Expression trend of differentially expressed genes (DEGs) involved in (a) Th1 and Th2, and (b) Th17 cell differentiation at the prepatent (AP), early (EP), peak (PP), and late (LP) oocyst-shedding periods. Genes marked red have up-regulated expression, while those marked blue have down-regulated expression.

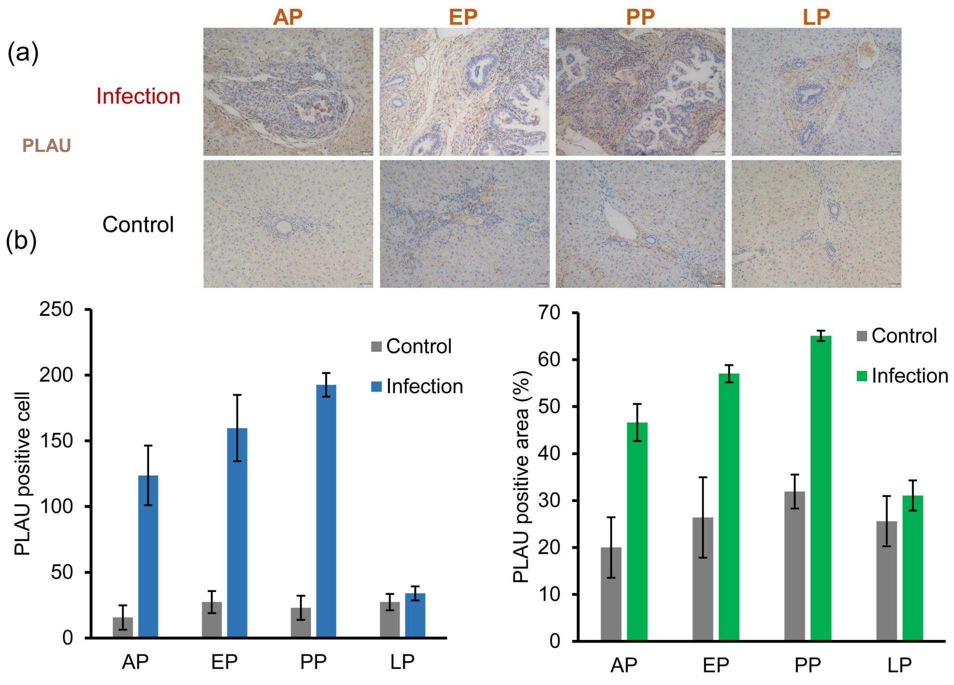

Figure S6: Expression changes of PLAU at the prepatent (AP), early (EP), peak (PP), and late (LP) oocyst-shedding periods, based on immunohistochemistry. (a) Immunohistochemistry analysis of PLAU expression in the liver of rabbits collected at different periods. Scale bars,50 µm. The (b) number and (c) percentage of positive cells and were quantified using the Image J software.

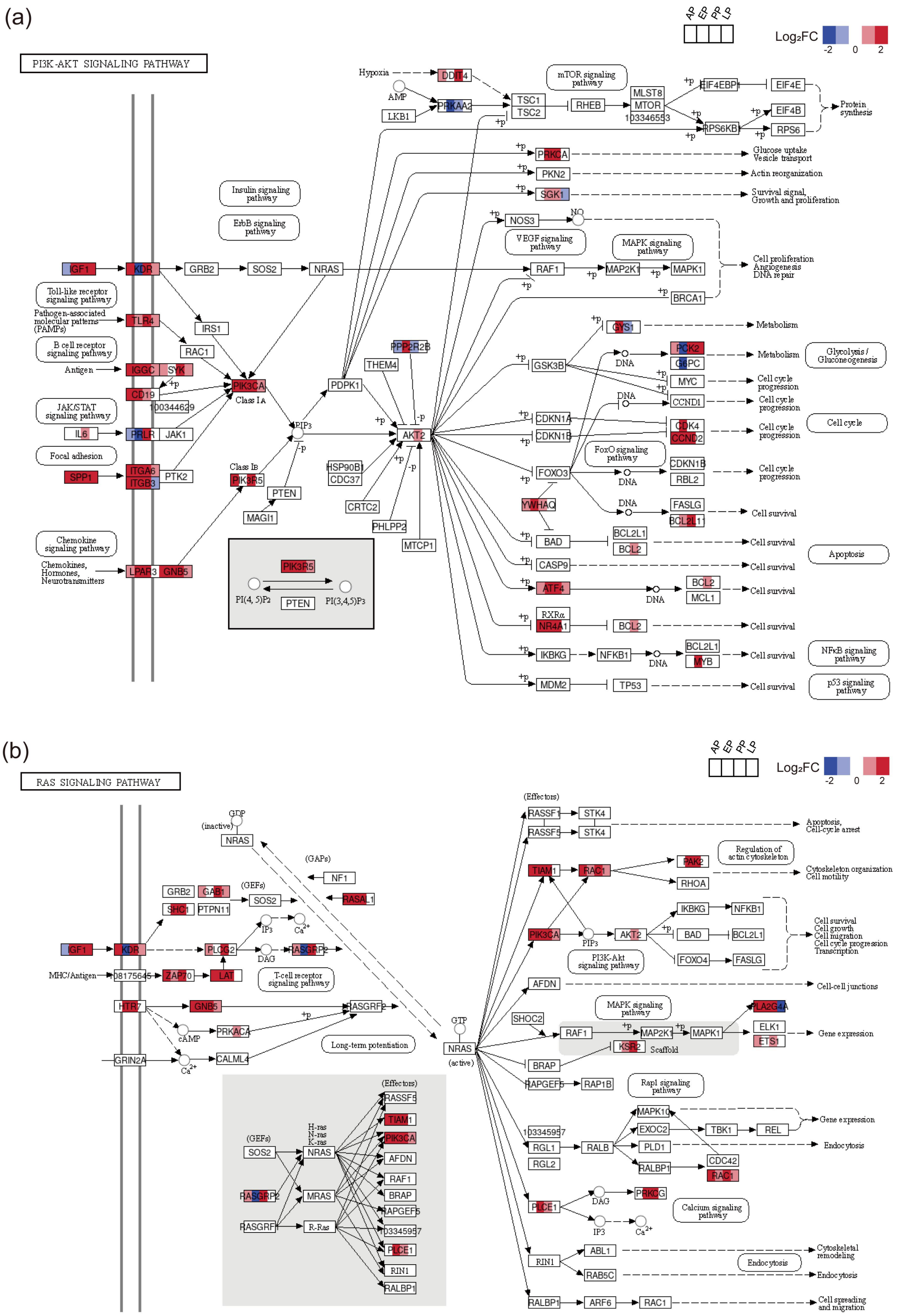

Figure S7: Transcriptomic changes of differentially expressed genes (DEGs) involved in the PI3K-Akt and Ras signaling pathways in the liver during *Eimeria stiedae* infection. Expression trend of DEGs involved in (a) PI3K-Akt and (b) Ras signaling pathways in the liver infected with *E. stiedae* at the prepatent (AP), early (EP), peak (PP), and late (LP) oocyst-shedding periods. Genes marked red have up-regulated expression, while those marked blue have down-regulated expression.

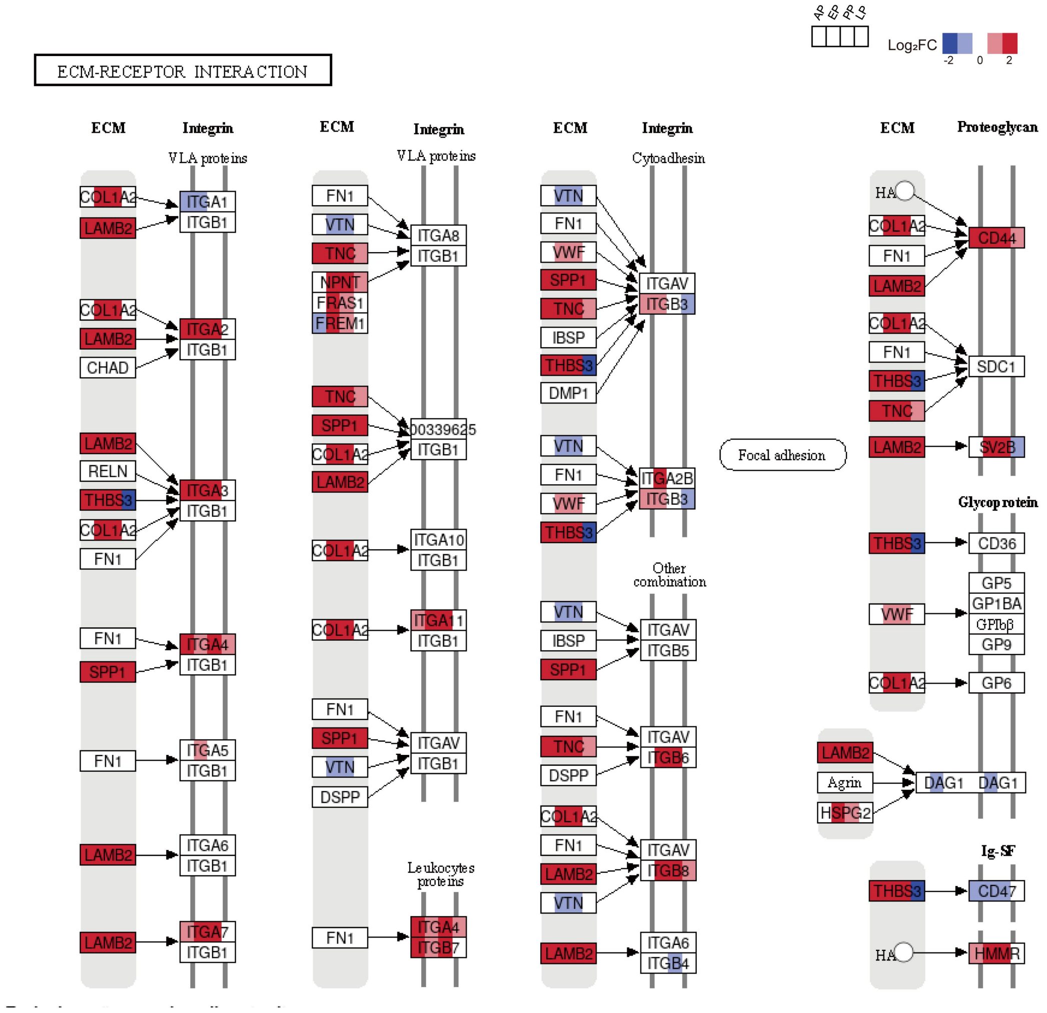

Figure S8: Expression changes of differentially expressed genes (DEGs) involved in the ECM-receptor interaction in the liver at the prepatent (AP), early (EP), peak (PP), and late (LP) oocyst-shedding periods. Genes marked red have up-regulated expression, while those marked blue have down-regulated expression.

Supplementary Table

Table S1: Primers used in qRT-PCR to validate the RNA-seq results.

Gene	Primer	Sequence (5'-3')
GAPDH	Forward	GGCGTGAACCACGAGAAGTA
	Reverse	TCCACAATGCCGAAGTGGTC
STAT6	Forward	AACGCTGCCTCAGGAGCTAC
	Reverse	CTGAAGCGGAGGAGGAAGGT
IL4R	Forward	TTACCTGGCAGGACCAGTAT
	Reverse	GTCACGTTGACGCTGAAGAT
STAT4	Forward	CGTGGCCTTAACTCAGATCA
	Reverse	CAAGCCATGTCCAGAAGGTA
IL1B	Forward	TGTTGTCTGGCACGTATGAG
	Reverse	GCCACAGGTATCTTGTCGTT
TLR4	Forward	GCCATAGCTTCTTGCATGTC
	Reverse	TGTCTCCACAGCCACTAGTT

Table S2: Summary of sequencing reads mapping to the reference genome and quality parameters.

Period	Group	Sample	Raw data			Clean data		Mapped reads with *O. cuniculus* genome		Mapped reads with *E. stiedai* genome	
			Data size (Gb)	No. of reads	GC (%)	Clean reads (%)	GC (%)	Total mapped reads	Mapped (%)	Total mapped reads	Mapped (%)
AP	Infection	i1	7.0	46,349,632	53	94.8	52	19,282,862	87.78	140,243	0.64
		i2	7.1	47,263,294	52	96.7	52	20,193,284	88.32	34,793	0.15
		i5	7.1	47,266,506	52	97.0	52	19,893,454	86.80	277,463	1.21
		i6	6.8	45,278,064	52	96.8	52	19,553,969	89.21	2,778	0.01
	Control	c7	6.2	41,577,854	52	96.6	51	18,064,077	89.93	2,450	0.01
		c8	6.9	45,766,932	52	96.0	52	19,794,215	90.09	1,540	0.01
		c9	6.9	45,760,450	52	95.7	52	19,430,511	88.73	1,811	0.01
		c10	6.9	46,022,558	53	95.9	52	19,505,395	88.38	1,981	0.01
EP	Infection	i1	6.2	41,604,168	52	98.8	52	16,014,583	77.66	1,390,849	6.74
		i2	6.7	44,697,346	52	98.5	52	17,703,764	79.89	648,528	2.93
		i3	6.9	46,034,938	52	98.5	52	17,138,740	74.99	1,403,140	6.14
		i4	7.0	46,771,434	50	98.5	50	18,764,859	80.90	524,249	2.26
	Control	c5	7.4	49,110,076	51	98.9	51	20,903,557	85.74	862	0.00
		c6	7.2	48,222,758	51	98.3	51	19,757,359	82.67	670	0.00

PP	Infection	c7	6.7	44,422,874	50	98.8	50	18,763,839	85.06	1,022	0.00
		c8	6.8	45,659,696	51	98.9	51	19,439,852	85.74	868	0.00
		i2	7.4	49,027,014	53	98.7	52	17,885,812	73.46	2,549,652	10.47
		i4r	6.5	43,300,852	51	99.3	51	17,410,923	80.80	1,330,254	6.17
		i6r	7.1	47,551,910	51	99.0	51	19,054,565	80.53	976,727	4.13
		i4	7.1	47,446,270	52	99.2	51	18,585,130	78.89	1,505,214	6.39
	Control	c8r	7.0	46,953,518	51	99.3	51	20,328,659	87.04	803	0.00
		c9r	7.0	46,885,420	52	99.0	51	20,068,917	86.13	740	0.00
		c10r	7.1	47,592,664	51	99.2	51	20,507,189	86.64	692	0.00
		c12r	7.0	46,418,448	51	99.0	51	19,673,916	85.23	406	0.00
LP	Infection	i2	7.7	51,507,862	50	98.3	50	20,901,640	81.49	1,290	0.01
		i5	7.3	48,950,830	50	98.4	50	19,595,824	80.39	1,276	0.01
		i6	7.6	50,662,962	51	98.4	51	20,382,514	80.80	1,293	0.01
	Control	c7	7.7	51,545,316	50	98.4	50	20,834,512	81.20	2,377	0.01
		c8	7.2	47,725,270	51	98.9	51	19,669,884	82.80	886	0.00
		c9	7.4	49,098,448	51	98.7	51	20,036,612	81.94	1,440	0.01

Note: AP, the prepatent/asexual oocyst-shedding period. EP, the early oocyst-shedding period. PP, the peak oocyst-shedding period. LP, the late oocyst-shedding period.

Table S3: KEGG enrichment among the up-regulated genes in different periods.

	AP	EP	PP	LP
Numbers of up-regulated genes (Fold Change ⩾ 2)	794	1870	1923	164
Numbers of KEGG pathways (p ＜0.05)	79	60	116	12
Numbers of immune-related pathways	25	9	25	0
Ratio of immune-related pathways	31.60%	15.00%	21.60%	0%

Note: AP, the prepatent/asexual oocyst-shedding period. EP, the early oocyst-shedding period. PP, the peak oocyst-shedding period. LP, the late oocyst-shedding period.

Table S4. Summary of STEM significantly clustered profiles.

	Term	Count	Gene ratio	*P* Value	FDR
Profile 5	ocu04146:Peroxisome	25	3.293808	4.1E-14	5E-12
	ocu01240:Biosynthesis of cofactors	28	3.689065	2.8E-10	2E-08
	ocu00071:Fatty acid degradation	16	2.108037	5.8E-10	3E-08
	ocu00040:Pentose and glucuronate interconversions	13	1.71278	1.3E-08	6E-07
	ocu00830:Retinol metabolism	18	2.371542	3.9E-08	1E-06
	ocu00053:Ascorbate and aldarate metabolism	12	1.581028	7.2E-08	2E-06
	ocu00280:Valine, leucine and isoleucine degradation	15	1.976285	9.2E-08	3E-06
	ocu00140:Steroid hormone biosynthesis	16	2.108037	9.8E-07	2E-05
	ocu00980:Metabolism of xenobiotics by cytochrome P450	16	2.108037	9.8E-07	2E-05
	ocu00620:Pyruvate metabolism	13	1.71278	1E-06	2E-05
	ocu04976:Bile secretion	17	2.239789	1.1E-06	2E-05
	ocu00630:Glyoxylate and dicarboxylate metabolism	11	1.449275	1.5E-06	2E-05
	ocu01212:Fatty acid metabolism	13	1.71278	4.5E-06	7E-05
	ocu04979:Cholesterol metabolism	11	1.449275	3.1E-05	4E-04
	ocu00860:Porphyrin metabolism	10	1.317523	9.1E-05	0.001
	ocu00120:Primary bile acid biosynthesis	7	0.922266	0.00011	0.001
	ocu04977:Vitamin digestion and absorption	8	1.054018	0.00011	0.001
	ocu01200:Carbon metabolism	16	2.108037	0.00018	0.002
	ocu00350:Tyrosine metabolism	8	1.054018	0.00064	0.006
	ocu00380:Tryptophan metabolism	9	1.185771	0.0011	0.01
	ocu00790:Folate biosynthesis	7	0.922266	0.0013	0.011
	ocu00410:beta-Alanine metabolism	7	0.922266	0.0013	0.011
	ocu03320:PPAR signaling pathway	11	1.449275	0.0014	0.011
	ocu04714:Thermogenesis	21	2.766798	0.00233	0.018
	ocu04610:Complement and coagulation cascades	10	1.317523	0.00412	0.03
	ocu00310:Lysine degradation	9	1.185771	0.0056	0.039

	ocu00650:Butanoate metabolism	6	0.790514	0.00753	0.051
	ocu00230:Purine metabolism	13	1.71278	0.00843	0.055
	ocu00260:Glycine, serine and threonine metabolism	7	0.922266	0.01249	0.08
	ocu00760:Nicotinate and nicotinamide metabolism	6	0.790514	0.01307	0.081
	ocu05012:Parkinson disease	21	2.766798	0.01642	0.099
	ocu05207:Chemical carcinogenesis - receptor activation	16	2.108037	0.02329	0.137
	ocu04936:Alcoholic liver disease	12	1.581028	0.02553	0.146
	ocu00190:Oxidative phosphorylation	13	1.71278	0.03249	0.181
	ocu04975:Fat digestion and absorption	7	0.922266	0.03619	0.197
	ocu04710:Circadian rhythm	5	0.658762	0.0436	0.231
	ocu00220:Arginine biosynthesis	4	0.527009	0.04693	0.243
	ocu02010:ABC transporters	6	0.790514	0.04791	0.243
	ocu00670:One carbon pool by folate	4	0.527009	0.0535	0.265
	ocu00640:Propanoate metabolism	5	0.658762	0.05813	0.282
	ocu04964:Proximal tubule bicarbonate reclamation	4	0.527009	0.06048	0.287
	ocu04723:Retrograde endocannabinoid signaling	11	1.449275	0.06859	0.318
	ocu00010:Glycolysis / Gluconeogenesis	7	0.922266	0.0698	0.318
	ocu04931:Insulin resistance	9	1.185771	0.07503	0.335
	ocu00590:Arachidonic acid metabolism	8	1.054018	0.07921	0.346
	ocu05010:Alzheimer disease	23	3.030303	0.09769	0.419
Profile 16	ocu04512:ECM-receptor interaction	25	1.904037	1.6E-10	5E-08
	ocu04510:Focal adhesion	33	2.513328	1.6E-07	2E-05
	ocu04066:HIF-1 signaling pathway	21	1.599391	1.1E-05	0.001
	ocu04151:PI3K-Akt signaling pathway	40	3.046458	5.5E-05	0.002
	ocu04360:Axon guidance	26	1.980198	6.6E-05	0.002
	ocu04810:Regulation of actin cytoskeleton	28	2.132521	0.00025	0.007
	ocu00051:Fructose and mannose metabolism	9	0.685453	0.00073	0.016
	ocu04668:TNF signaling pathway	16	1.218583	0.0012	0.023
	ocu04514:Cell adhesion molecules	20	1.523229	0.0021	0.035
	ocu04060:Cytokine-cytokine receptor	30	2.284844	0.00231	0.036

interaction				
ocu04640:Hematopoietic cell lineage	15	1.142422	0.00334	0.048
ocu04970:Salivary secretion	13	0.990099	0.00443	0.061
ocu04926:Relaxin signaling pathway	17	1.294745	0.00477	0.062
ocu04972:Pancreatic secretion	16	1.218583	0.00522	0.065
ocu00520:Amino sugar and nucleotide sugar metabolism	9	0.685453	0.00767	0.088
ocu04015:Rap1 signaling pathway	22	1.675552	0.00935	0.099
ocu05145:Toxoplasmosis	16	1.218583	0.00961	0.099
ocu05414:Dilated cardiomyopathy	14	1.06626	0.01013	0.1
ocu05202:Transcriptional misregulation in cancer	21	1.599391	0.01061	0.101
ocu00052:Galactose metabolism	7	0.53313	0.01263	0.117
ocu04062:Chemokine signaling pathway	19	1.447068	0.01809	0.162
ocu04270:Vascular smooth muscle contraction	16	1.218583	0.01871	0.163
ocu04974:Protein digestion and absorption	14	1.06626	0.01963	0.164
ocu05100:Bacterial invasion of epithelial cells	11	0.837776	0.02006	0.164
ocu01250:Biosynthesis of nucleotide sugars	7	0.53313	0.02203	0.176
ocu04612:Antigen processing and presentation	10	0.761615	0.026	0.199
ocu05410:Hypertrophic cardiomyopathy	12	0.913938	0.02656	0.199
ocu04650:Natural killer cell mediated cytotoxicity	13	0.990099	0.02708	0.199
ocu04918:Thyroid hormone synthesis	10	0.761615	0.03066	0.219
ocu05332:Graft-versus-host disease	7	0.53313	0.03159	0.219
ocu05170:Human immunodeficiency virus 1 infection	21	1.599391	0.03203	0.219
ocu04659:Th17 cell differentiation	13	0.990099	0.03289	0.219
ocu00524:Neomycin, kanamycin and gentamicin biosynthesis	3	0.228484	0.03393	0.221
ocu04919:Thyroid hormone signaling pathway	13	0.990099	0.03501	0.223
ocu05169:Epstein-Barr virus infection	21	1.599391	0.0365	0.228
ocu04022:cGMP-PKG signaling pathway	17	1.294745	0.0435	0.266
ocu04930:Type II diabetes mellitus	7	0.53313	0.04802	0.287

	ocu04020:Calcium signaling pathway	22	1.675552	0.04993	0.292
	ocu00512:Mucin type O-glycan biosynthesis	6	0.456969	0.05086	0.292
	ocu00533:Glycosaminoglycan biosynthesis - keratan sulfate	4	0.304646	0.05186	0.292
	ocu04530:Tight junction	17	1.294745	0.05471	0.302
	ocu04911:Insulin secretion	10	0.761615	0.0588	0.318
	ocu04925:Aldosterone synthesis and secretion	11	0.837776	0.06182	0.329
	ocu04144:Endocytosis	22	1.675552	0.06457	0.337
	ocu01230:Biosynthesis of amino acids	9	0.685453	0.0718	0.357
	ocu04371:Apelin signaling pathway	13	0.990099	0.07185	0.357
	ocu04350:TGF-beta signaling pathway	11	0.837776	0.07341	0.357
	ocu04625:C-type lectin receptor signaling pathway	11	0.837776	0.07341	0.357
	ocu05330:Allograft rejection	7	0.53313	0.07483	0.358
	ocu04657:IL-17 signaling pathway	10	0.761615	0.07992	0.376
	ocu04922:Glucagon signaling pathway	11	0.837776	0.08184	0.379
	ocu04014:Ras signaling pathway	20	1.523229	0.08347	0.38
	ocu04940:Type I diabetes mellitus	7	0.53313	0.08752	0.387
	ocu04658:Th1 and Th2 cell differentiation	10	0.761615	0.08951	0.387
	ocu04064:NF-kappa B signaling pathway	11	0.837776	0.09084	0.387
	ocu04660:T cell receptor signaling pathway	11	0.837776	0.09084	0.387
	ocu04024:cAMP signaling pathway	19	1.447068	0.09173	0.387
Profile 14	ocu04110:Cell cycle	27	4.856115	5.4E-17	1E-14
	ocu03030:DNA replication	13	2.338129	6.5E-12	7E-10
	ocu04114:Oocyte meiosis	14	2.517986	5.4E-06	4E-04
	ocu04914:Progesterone-mediated oocyte maturation	10	1.798561	0.00028	0.013
	ocu03440:Homologous recombination	7	1.258993	0.00036	0.013
	ocu00240:Pyrimidine metabolism	8	1.438849	0.00046	0.015
	ocu03410:Base excision repair	6	1.079137	0.00068	0.019
	ocu04540:Gap junction	9	1.618705	0.00092	0.023
	ocu03430:Mismatch repair	5	0.899281	0.00172	0.038
	ocu04145:Phagosome	12	2.158273	0.00204	0.041

	ocu04514:Cell adhesion molecules	24	4.316547	8.8E-12	9E-10
	ocu04640:Hematopoietic cell lineage	19	3.417266	2.3E-10	1E-08
	ocu04672:Intestinal immune network for IgA production	15	2.697842	2.2E-09	9E-08
	ocu05330:Allograft rejection	13	2.338129	1.3E-08	3E-07
	ocu04658:Th1 and Th2 cell differentiation	15	2.697842	6.8E-08	1E-06
	ocu04145:Phagosome	19	3.417266	2.7E-07	3E-06
	ocu05321:Inflammatory bowel disease	13	2.338129	3.1E-07	4E-06
	ocu04660:T cell receptor signaling pathway	15	2.697842	3.9E-07	4E-06
	ocu04380:Osteoclast differentiation	15	2.697842	2.4E-06	3E-05
	ocu04611:Platelet activation	14	2.517986	1.4E-05	1E-04
	ocu04612:Antigen processing and presentation	11	1.978417	1.5E-05	1E-04
	ocu04060:Cytokine-cytokine receptor interaction	21	3.776978	3.7E-05	3E-04
	ocu04072:Phospholipase D signaling pathway	15	2.697842	5.5E-05	5E-04
Profile 11	ocu04664:Fc epsilon RI signaling pathway	10	1.798561	0.0001	8E-04
	ocu04062:Chemokine signaling pathway	15	2.697842	0.00018	0.001
	ocu04659:Th17 cell differentiation	12	2.158273	0.0002	0.001
	ocu04666:Fc gamma R-mediated phagocytosis	11	1.978417	0.00036	0.002
	ocu04620:Toll-like receptor signaling pathway	10	1.798561	0.00057	0.004
	ocu04610:Complement and coagulation cascades	9	1.618705	0.00075	0.005
	ocu04151:PI3K-Akt signaling pathway	20	3.597122	0.00136	0.008
	ocu04662:B cell receptor signaling pathway	8	1.438849	0.00322	0.017
	ocu04725:Cholinergic synapse	9	1.618705	0.00447	0.024
	ocu04613:Neutrophil extracellular trap formation	13	2.338129	0.00492	0.025
	ocu04014:Ras signaling pathway	14	2.517986	0.00666	0.033
	ocu04064:NF-kappa B signaling pathway	9	1.618705	0.00673	0.033
	ocu04010:MAPK signaling pathway	16	2.877698	0.00875	0.041

ocu04913:Ovarian steroidogenesis	6	1.079137	0.01063	0.047
